# What physicians want to learn about sickness certification: analyses of questionnaire data from 4019 physicians

**DOI:** 10.1186/1471-2458-10-61

**Published:** 2010-02-09

**Authors:** Anna Löfgren, Jan Hagberg, Kristina Alexanderson

**Affiliations:** 1 Division of Insurance Medicine, Department of Clinical Neuroscience, Karolinska Institutet, S-171 77 Stockholm, Sweden

## Abstract

**Background:**

Sickness absence is a problem in many Western countries. Physicians have an essential role in sickness certification of patients, which is often recommended in health care but may have side effects. Despite the potentially harming impact of sickness absence, physicians have very limited training in insurance medicine, and there is little research on sickness certification practices. Our aim was to ascertain what knowledge and skills physicians in different clinical settings feel they need in order to improve their competence in sickness certification.

**Methods:**

The data for analysis were collected in 2004 in Stockholm and Östergötland Counties, Sweden, by use of a comprehensive questionnaire about sickness certification issues, which was sent to 7,665 physicians aged ≤ 64 years. The response rate was 71% (n = 5455). Analyses of association and factor analysis were applied to the various aspects of competence to establish a skills index and a knowledge index, which were used to compare the results for physicians in different clinical settings.

**Results:**

Most physicians stated they needed more knowledge and skills in handling sickness certification, e.g. regarding how to assess work capacity (44%) and optimal length and degree of sickness absence (50%), and information about aspects of the social insurance system (43-63%). Few (20%) reported needing to know more about issuing sickness certificates. The index scores varied substantially between different clinical settings, and this disparity remained after adjustment for sex, years in practice, workplace policy, and support from management. Scores on the skills index were significantly higher for physicians in primary care than for those working in other areas.

**Conclusions:**

A majority of physicians in most types of clinics/practices, not only primary care, indicated the need for more knowledge and skills in handling sickness certification cases. Increased knowledge and skills are needed in order to protect both the health and equity of patients. However, few physicians stated that they needed more skills in filling out sickness certificates, which contradicts previous findings about such documents being of poor quality and suggests that factors other than mere knowledge and skills are involved.

## Background

Physicians have a key role in the sickness insurance systems in most countries. In Sweden, these medical professionals are responsible for providing the Social Insurance Office (SIO) with sickness certificates for patients who are unable to work as a result of disease or injury. Thus in patient consultations, the physician is to perform seven specific tasks [1]: (1) establish a diagnosis; (2) assess functional limitations and work capacity; (3) discuss with the patient the pros and cons of being off sick; (4) determine duration and degree of sick leave (i.e. full or part time [2]); (5) together with the patient, plan possible measures to be taken during the absence, including treatment and/or rehabilitation (6) fill out a sickness certificate for the employer and the SIO; (7) document these decisions, measures, and plans. The amount of training that physicians receive in insurance medicine varies between different countries [3]. In Sweden, physicians have only limited pre- and postgraduate education in this area, which is in fact not a specialty in its own, in contrast to what is seen in some other countries. Furthermore, continuing professional development (CPD) in insurance medicine in Sweden is not compulsory, is often restricted to just a few hours of actual training, and is usually organised by the Swedish Social Insurance Agency, not by academic institutions.

Moreover, most CPD activities in sickness insurance have been directed towards general practitioners (GPs). However, we [4] recently found that although 62% of GPs had consultations involving sickness certification more than five times a week, physicians in many other clinical settings also used a substantial part of their daily work time to manage this task, and in some cases they did so even more often than GPs, as exemplified by the finding that 83% of the orthopaedics had such consultations more than five times a week.

Notably, a systematic review of the literature has also provided evidence that physicians frequently find it problematic to handle sickness certification tasks and that the sick notes they issue are often of low quality [1]. Furthermore, similar conclusions have been drawn in studies that were published after that review [4-11]. Problems with sickness certification have been found to be common among physicians in many different clinical workplaces, but most often by GPs, 60% of whom were noted to experience such difficulties at least every week, most frequently in the form of handling disagreements with patients [4]. Previous investigations have also shown that physicians feel that they lack competence in insurance medicine [6] to such an extent that it is troublesome in the health care system and causes sickness certification tasks to be regarded as a psychosocial work problem [7]. Moreover, recent studies have revealed deficiencies in physicians' competence related to assessment of work capacity [8,10,12,13] and in communication skills [10]. Notwithstanding, to achieve optimal training in insurance medicine, it is also necessary to know what physicians themselves want to learn in order to be able to design interventions that can benefit these professionals. Searching the literature, we found no previous investigations on that subject. Thus, *our aim *was to ascertain what knowledge and skills physicians in different clinical settings feel they need in order to improve their competence in sickness certification.

## Methods

In 2004, we conducted a questionnaire study including 7665 physicians < 65 years of age in Stockholm and Östergötland Counties in Sweden [4]. Those individuals represented 24% of all physicians in Sweden in that age group [14].

### Study population

In Stockholm, data were collected from all members of the Swedish Medical Association (SMA) aged ≤ 64 years who were registered as practising or living in Stockholm County (the largest county in Sweden) in 2004. At that time about 95% of all physicians in Sweden were members of the SMA. All types of physicians in Stockholm County were included in the survey, because the SMA register did not compile information about specialties or type of workplaces.

In Östergötland County, the local branch of the SMA did not permit access to their register, and thus, to identify participants, we used a register of all physicians in Sweden compiled by the private company Pharma Marketing AB. We selected all physicians aged ≤ 64 years and working in Östergötland County, with the exception of those employed at clinics/practices dealing very little with sickness certification (e.g. geriatrics and paediatrics) and those who were registered to work in the county but were living abroad or not currently practising as physicians.

### The questionnaire

A comprehensive 96-item questionnaire about sickness certification was developed [4], and detailed information about construction and administration of the instrument have been reported elsewhere [4]. The items included were based on the results of previous studies [1,6] and on discussions with reference groups, researchers, and teachers in the field. The questionnaire was tested in a pilot study in June 2004 (n = 102), which led to minor revisions.

In October 2004, the questionnaire was mailed to the participants, with two reminders. When available, the physicians' home addresses were used (96%) to avoid interaction with colleagues during completion. To guarantee the anonymity of the participants, this process was conducted by Statistics Sweden, and the authors later received anonymised data. The study was approved by the Regional Ethical Review Board of Stockholm, Sweden.

The overall response rate was 71% (Table 1), and the rates in the two counties were similar (71% and 72%). The proportion of physicians reporting having consultations concerning sickness certification was larger in Östergötland than in Stockholm County (91% and 71%, respectively), which was due to the above-mentioned differences in selection of the participants, where geriatrics and paediatrics were not included in Östergötland. The present analyses included only data from the 4,019 physicians (74%) who reported that they dealt with sickness certification at least a few times a year (Table 1).

**Table 1:** Response rate and characteristics of the physicians in the study population

	Study population	Respondents		Respondents having consultations concerning sickness certification at least a few times a year	
			
	n	n	% of study population	n	% of the respondents
All	7665	5455	71	4019	74
					
*County*					
Stockholm	6794	4827	71	3446	71
Östergötland	871	628	72	573	91
					
*Sex*					
Women	3646	2710	74	2007	74
Men	4019	2745	68	2012	73
					
*Educational level*^1^					
Non-specialist		1381		1046	76
Specialist		3995		2947	74
No information		79		26	33
					
*Mean age *(years)	48.2	48.3		47.7	

^1^Information on educational level was not available for the study population

### Data analyses

The questionnaire included 14 items about the need for CPD, and the answers to those items were analysed in general and regarding associations with demographic and work-related factors. The phrasings of the CPD items and response options are presented in Table 2. The stem of the questions read: "To what extent do you need further knowledge and skills concerning the following? Please respond to each of the suggestions (a-n)." Work-related items covered the following:

- years practicing medicine (How many years have you practiced medicine since you got your medical degree?);

- educational level (What is your highest level of medical education? Medical degree/Registered physician/In resident training/Specialist);

- frequency of consultations (How often in your daily work do you have consultations including consideration of sickness certification? A few times a year/About once a month/1-5 times a week/6-20 times a week/More than 20 times a week);

- frequency of problems (How often in your daily work do you find it problematic to handle sickness certification? Never, almost never/A few times a year/About once a month/1-5 times a week/6-20 times a week/More than 20 times a week);

- contacts with the SIO (Do you have regular, scheduled contacts with the SIO, e.g. coordinating meetings, rehabilitation meetings, sickness certification committee conferences, and/or SIO officers at your practice? Yes, to a satisfactory degree/Yes, but would prefer more extensive contact/No, but would like to/No, and satisfied not to);

- support from management (Do you have support from management regarding handling of sickness certification cases? Yes, to a large degree/Yes, to some degree/No);

- workplace policy (Do you at your clinic/practice have a common strategy for handling matters related to sickness certification? Yes, and it is well established/To some extent/No).

**Table 2:** How physicians rate their need for the different items regarding continuing professional development in sickness certification.

			Large need	Fairly large need	Limited need	No need	All
		
Item^1^		n	%	%	%	%	%
N	The options and responsibilities of the Employment Office in sickness certification cases	3861	20.8	41.9	26.8	10.6	100
M	Employers' options and responsibilities in sickness certification cases	3871	19.0	43.4	28.3	9.3	100
L	The options and responsibilities of the SIO^2 ^in sickness certification cases	3871	18.3	43.4	29.0	9.2	100
I	Other forms of compensation in the social insurance system	3863	17.7	41.5	28.8	12.0	100
K	My options and responsibilities as a physician in sickness certification cases	3876	14.7	39.1	35.6	10.6	100
C	Assessing the optimum length and degree of sickness certification	3860	14.6	35.2	36.8	13.3	100
A	Judging patients' work capacity	3874	13.1	31.1	40.2	15.6	100
D	Handling conflicts with patients about the need for sickness certification	3864	12.7	26.7	41.7	18.9	100
B	Work demands in different occupations and/or workplaces	3855	12.0	33.8	40.0	14.2	100
H	Sickness insurance rules	3871	11.4	37.6	38.2	12.8	100
J	Private and supplementary insurance that patients often have	3861	11.2	31.5	38.8	18.5	100
F	Designing optimum plans of action	3857	8.5	33.7	37.9	19.9	100
G	Deciding when it is necessary to contact the SIO^2^	3857	4.3	24.5	48.1	23.1	100
E	Writing sickness certificates	3874	3.8	15.9	52.7	27.5	100

^1^Letters given to the items according to order of appearance in the questionnaire

^2^Social Insurance Office

Items sorted according to highest and lowest after percentage perceiving large need.

Responders were also asked to indicate which of eleven types of clinic or practice represented their main workplace. Information about age was retrieved from the SMA and Pharma Marketing AB registers. In some analyses, five different types of workplace were included, and physicians working in all other clinical settings served as a reference group. The five types of clinics/practices were primary care, psychiatry, occupational health, orthopaedics, and surgery, which were chosen to achieve variation in the number of sickness certification cases, levels of problems, and proportions of physicians working at hospitals, in primary care, and at occupational health services.

### Statistics

Descriptive statistics were calculated for the whole group. In addition, two separate analyses were used to discern associations between different CPD items to be able to establish overall indices. Initially, Kendall's tau-b was used to analyse associations, and items found to have tau-b values > 0.500 with two or more other items were regarded as having a moderately strong association. In the same way, items with only one or no value > 0.500 were regarded as showing insufficient associations. This analysis resulted in the identification of two separate factors. To confirm the results, we performed a factor analysis based on the Kendall's tau-b, which identified the same factors. Thus, the analysis of association (confirmed by the factor analyses), yielded two overall indices comprising five and six items, respectively. These indices could reach a maximum of 20 and 24, and were therefore presented as percentages of maximum to permit comparison of the index values. The use of percentages instead of absolute values does not affect the significance levels in the regression analysis. Respondents with missing values on all items in one index were not included in the analysis of that index. Partially missing values in the indices were set to zero. In the analyses of variance and the regression analyses, the partial non-response was 2.8-12.1% (median 3.8%).

Both one- and two-way univariate analysis of variance (ANOVA) and simple univariate and multiple linear regressions were used to evaluate the indices. The items were analysed separately and as parts of four different models. The variable "years practicing medicine" was used as a measure of the length of experience each physician had in performing sickness-certification tasks, because, compared to "age" and "educational level," it had the highest adjusted R^2 ^in linear regression conducted using the knowledge index and the skills index as dependent variables. Two items were used as measures of workplace support: "workplace policy" and "support from management." All data were used in those analyses, that is, none of the responses to the variables were dichotomised.

## Results

Half of the physicians (54%) indicated a large or fairly large need for further knowledge about their own options and responsibilities in the sickness certification process, and even more of these professionals (59-63%) reported needing additional information about other stakeholders involved in the process (Table 2). Many physicians also wanted further knowledge and skills to aid assessments of length and degree of sick leave (50%) and work demands in different occupations (46%). (It is possible to be on sick leave for part time in Sweden [2].) In contrast, few physicians reported a large or fairly large need in relation to the items "writing sickness certificates" (20%) and "deciding when it is necessary to contact the SIO" (29%).

In the analysis of associations between different CPD items, two groups emerged, which included six items concerning knowledge (H, I, K, L, M, and N) and five items about skills (A, B, C, D, and F), respectively (Table 3). All items in each group had at least two associations > 0.500 with other items in the same group, and no item had any associations > 0.500 with items in the other group. These results were confirmed by our factor analysis, or, in other words, the same two groups of items emerged in both types of assessment. Based on those two groups, we constructed two indices, which we designated the knowledge index and the skills index (Table 4). The response options were coded as follows: large need = 4, fairly large need = 3, limited need = 2, no need = 1, and missing = 0. Thus the maximum score that could be reached was 24 for the knowledge index and 20 for the skills index. The results obtained using these indices are given in percentages in order to facilitate comparison (Table 5). Item E showed no correlation (> 0.500) with any of the other items, and weak associations were found for items G and J, these three were therefore not included in the indices (Table 3).

**Table 3:** Results of analysis of associations (Kendall's tau-b) between items in the questionnaire^1^

Item	A^2^	B	C	D	E	F	G	H	I	J	K	L	M	N
A^2^	1.00	**0.68**	**0.70**	**0.50**	0.39	**0.56**	0.45	0.40	0.38	0.30	0.44	0.43	0.43	0.43
B		1.00	**0.63**	0.47	0.35	**0.52**	0.42	0.39	0.39	0.36	0.42	0.43	0.46	0.45
C			1.00	**0.53**	0.41	**0.55**	0.46	0.42	0.39	0.32	0.49	0.46	0.46	0.45
D				1.00	0.39	0.47	0.44	0.39	0.35	0.33	0.44	0.41	0.41	0.40
E					1.00	0.48	0.49	0.44	0.35	0.30	0.44	0.39	0.37	0.33
F						1.00	**0.57**	0.44	0.46	0.38	0.44	0.47	0.48	0.49
G							1.00	0.50^3^	0.43	0.38	0.47	0.46	0.45	0.43
H								1.00	**0.61**	0.43	**0.60**	**0.57**	**0.55**	**0.50**
I									1.00	**0.56**	**0.51**	**0.56**	**0.55**	**0.55**
J										1.00	0.42	0.43	0.44	0.46
K											1.00	**0.72**	**0.65**	**0.59**
L												1.00	**0.80**	**0.75**
M													1.00	**0.82**
N														1.00

^1^All associations were significant at the 0.001 level (2-tailed). Correlations > 0.500 shown in bold type

^2^See Table 4 or 2 for the content of the items A to N

^3^Rounded up from 0.497

**Table 4:** Items included in the two indices, coding of response alternatives, and mean for the whole group

	Knowledge Index (6 items)			Skills Index (5 items)	
***4 = large need***	***3 = fairly large need***	***2 = limited need***		***1 = no need***	***0*^1 ^= *missing value***

H	Sickness insurance rules	0-4	A	Judging patients' work capacity	0-4
I	Other forms of compensation in the social insurance system	0-4	B	Work demands in different occupations and/or workplaces	0-4
K	My options and responsibilities as a physician in sickness certification cases	0-4	C	Assesing the optimum length and degree of sickness certification	0-4
L	The options and responsibilities of the SIO^2 ^in sickness certification cases	0-4	D	Handling conflicts with patients about the need for sickness certification	0-4
M	Employers' options and responsibilities in sickness certification cases	0-4	F	Designing optimum plans of action	0-4
N	The options and responsibilities of the Employment Office in sickness certification cases	0-4			
					
	Sum^1^	1-24		Sum^1^	1-20
	Unadjusted mean	15.7		Unadjusted mean	11.9
	Unadjusted mean percent of maximum	**65.6%**		Unadjusted mean percent of maximum	**59.4%**

^1^Respondents with missing values on all items in one index were not included in the analysis of that index

^2^Social Insurance Office

**Table 5:** Demographic and work-related items associated with expressed need for skills and knowledge.

	Knowledge-index^1^				Skills-index^2^			
	
	n	group mean/reference group mean	difference from reference group mean	p	n	group mean/reference group mean	difference from reference group mean	p
*All*	3900	65.6			3905	59.4		
								
*Sex *(ref: male)	1961	62.8			1966	57.5		
Female	1939		+5.5	<0.001	1939		+3.7	<0.001
								
*Educational level *(ref: specialist)	2850	63.6			2856	56.8		
Medical degree	187		+10.0	<0.001	186		+14.3	<0.001
Registered physician	121		+7.1	<0.001	121		+9.1	<0.001
In resident training	717		+6.9	<0.001	717		+8.5	<0.001
								
*Main workplace*								
Psychiatry	348	72.6			347	63.5		
Primary health care centre	952	71.4			952	71.1		
Oncology	103	67.5			102	56.0		
Addiction medicine	38	66.9			38	58.2		
Internal medicine	359	66.2			359	59.7		
Orthopaedics	183	65.1			184	55.9		
Rehabilitation care^3^	58	62.5			58	56.6		
Other^4^	1152	62.0			1158	53.2		
Gynaecology/Obstetrics	278	61.7			278	54.2		
Occupational health services	122	59.8			122	58.5		
Surgery	290	57.2			290	49.6		
								
*Support from management *(ref: yes)	982	64.8			982	58.6		
No	2761		+1.6	0.018	2763		+1.7	0.013
								
*Workplace policy *(ref: yes)	564	57.9			564	51.6		
No	3256		+9.2	<0.001	3259		+9.4	<0.001
								
*Frequency of sickness certification cases *(ref: low frequency)	1925	63.3			1929	56.7		
High frequency	1975		+4.5	<0.001	1976		+5.3	<0.001
								
*Frequency of problems in handling sickness certification *(ref: low frequency)	3603	65.1			3607	58.6		
High frequency	251		+7.2	<0.001	252		+12.0	<0.001
								
*Regular contacts with SIO*^5 ^(ref: yes)	3024	68.5			3024	67.3		
No	841		-3.8	<0.001	844		-10.0	<0.001

^1^Sickness insurance rules, other forms of compensation and options and responsibilities of the physician, the SIO, the employer, and the employment office

^2^Judging work capacity, assess work demands, assess optimum length and degree of certification, handling conflicts, designing plans of action

^3^Including physicians in social insurance office and insurance company

^4^All other types of workplaces

^5^Social Insurance Office

One-way ANOVA. Mean index value, in percent of maximum.

According to the analyses presented in Table 5, the need for knowledge was slightly higher than the need for skills, with unadjusted means of maximum values at 65.6% and 59.4%, respectively. The gender-related difference was significant but small, with 5.5% and 3.7% higher means for women. The means decreased 0.4% (p < 0.001) and 0.5% (p < 0.001) for each year of age, and 0.4% (p < 0.001) and 0.5% (p < 0.001) for each year in practice.

Compared to specialists, non-specialists reported greater need for both knowledge and skills. The need for knowledge and skills differed very little between the physicians who did and those who did not have support from management. Physicians working at clinics/practices with a workplace policy for handling sickness certification cases had lower need for further knowledge and skills. Physicians working in psychiatry expressed more need for *knowledge *than all other groups, whereas those in primary care reported a greater need for *skills *compared to all others (Table 5).

Multiple regression analysis comparing physicians working in five different clinical settings (primary care, psychiatry, occupational health services, orthopaedics, and surgery) with physicians working in any of all other types of clinics/practices (Table 6) showed that the need for both skills and knowledge was significantly higher for physicians in primary care and psychiatry but significantly lower for those in surgery. Despite adjustment for four separate items (sex, years in practice, workplace policy, and support from management), the indicated differences remained, i.e. they resembled the crude values (model 4, Table 6).

**Table 6:** Crude and adjusted measure of expressed needs for skills and knowledge in different clinics.

		Knowledge-index^1^				Skills-index^2^			
	
	*Worksite*	n	reference group mean	diff. from ref. mean	p	n	reference group mean	diff. from ref. mean	p
*Crude*	*Ref: physicians working in any of all other clinics or practices*	1988	63.1			1993	54.8		
	Primary health care centre	952		+8.3	<0.001	952		+16.2	<0.001
	Psychiatry	348		+9.4	<0.001	347		+8.7	<0.001
	Occupational health services	122		-3.3	0.053	122		+3.6	0.026
	Orthopaedics	183		+1.9	0.177	184		+1.0	0.447
	Surgery	290		-5.9	<0.001	290		-5.3	<0.001
									
*Model 1*	*Adjusted for sex (ref: physicians working in any of all other clinics or practices)*	1988	65.4			1993	56.1		
	Primary health care centre	952		+8.2	<0.001	952		+16.1	<0.001
	Psychiatry	348		+9.2	<0.001	347		+8.6	<0.001
	Occupational health services	122		-3.2	0.062	122		+3.7	0.023
	Orthopaedics	183		+3.5	0.015	184		+1.8	0.176
	Surgery	290		-4.6	<0.001	290		-4.6	<0.001
									
*Model 2*	*Adjusted for sex, years in practice (ref: physicians working in any of all other clinics or practices)*	1890	72.0			1895	64.1		
	Primary health care centre	917		+7.8	<0.001	917		+15.8	<0.001
	Psychiatry	332		+9.0	<0.001	330		+8.7	<0.001
	Occupational health services	116		-1.2	0.476	116		+6.2	<0.001
	Orthopaedics	170		+3.7	0.009	171		+2.3	0.088
	Surgery	275		-5.3	<0.001	275		-5.2	<0.001
									
*Model 3*	*Adjusted for sex, years in practice, workplace policy (ref: physicians working in any of all other clinics or practices)*	1840	64.4			1843	56.9		
	Primary health care centre	916		+7.6	<0.001	916		+15.5	<0.001
	Psychiatry	317		+9.0	<0.001	315		+8.9	<0.001
	Occupational health services	115		-0.1	0.963	115		+7.3	<0.001
	Orthopaedics	167		+2.8	0.053	168		+1.4	0.305
	Surgery	270		-5.9	<0.001	270		-5.7	<0.001
									
*Model 4*	*Adjusted for sex, years in practice, workplace policy, support from management (ref: physicians working in any of all other clinics or practices)*	1788	64.2			1791	56.4		
	Primary health care centre	906		+7.6	<0.001	906		+15.8	<0.001
	Psychiatry	302		+9.0	<0.001	300		+8.9	<0.001
	Occupational health services	111		-0.5	0.762	111		+7.1	<0.001
	Orthopaedics	163		+2.3	0.111	164		+1.4	0.308
	Surgery	262		-5.7	<0.001	262		-5.6	<0.001

^1^Sickness insurance rules, other forms of compensation and options and responsibilities of the physician, the SIO, the employer and the employment office

^2^Judging work capacity, assess work demands, assess optimum length and degree of certification, handling conflicts, designing plans of action

Univariate ANOVA and univariate multiple linear regression. Mean index value in percent of maximum.

For physicians practising psychiatry, orthopaedics, or surgery, the difference compared to the reference group mean was the same for both indices. For example, physicians working in surgery had 5.7% (knowledge) and 5.6% (skills) lower values than physicians in any of all other clinics/practices (Model 4, Table 6). For those working in primary care and occupational health services, differences from the reference group mean were greater in the skills index than in the knowledge index.

## Discussion

This study included 4019 physicians who regularly had patient consultations involving sickness certification, and the majority of these professionals indicated that they needed to develop their knowledge and skills in relation to the process of certifying sick leave. However, there was substantial variation between different clinical settings, even after adjustment for demographic and work-related factors. A larger percentage of physicians in primary care and psychiatry reported a need for more knowledge and skills compared to other categories of physicians. Also, specialists expressed less need than non-specialists with respect to learning more about sickness certification. Nevertheless, the majority of all physicians reported that they did need CPD, especially regarding the roles of different stakeholders in sickness certification and how to assess patients' work capacity and optimal length and degree of sickness absence.

Strengths of this study include the large study population, the high response rate, and the many detailed questionnaire items about the aspects of interest. Another strength is that physicians in many different clinical settings were included - not only GPs, psychiatrists and orthopaedics, as has been the case in the majority of previous studies of physicians' sickness certification [1,6,12,15,16]. To what extent the results can be generalised to the whole of Sweden is uncertain. However, the two counties that were included comprise both large rural areas and very populous urban areas (with 2.3 million of the 9.1 million inhabitants in the entire country). Also, the present study population represented 24% of all physicians in Sweden, and, inasmuch as the response rate was high, the results can probably be generalised to at least the southern and central parts of the country, where 85% of the population lives. The variations between different types of clinics/practices are probably larger than the disparities between different parts of the country. The variation in sick-leave rates between different regions in Sweden does not necessarily indicate that physicians differ regarding their need for CPD. Generalisation to physicians in other countries cannot be done without considering the disparities in social insurance systems and in medical education. Despite that, studies conducted in other nations have also indicated that physicians feel they need more knowledge in insurance medicine [1,6,13]. The physicians' sickness certification tasks are basically the same in different welfare nations, as well as over the last decades. Moreover, development of the questionnaire employed in the current investigation involved a large number of clinicians and researchers focused on the discipline of interest, which, along with the fact that we obtained responses from a considerable number of participants, has provided an extensive base for claiming face validity.

As in all questionnaire studies, the data we acquired merely reflect the respondents' own assessments (here regarding their need for competence in sickness certification), which means that we do not have information on their actual proficiency in the areas of interest. The physicians' self-assessments in this context covered two aspects: (1) the competence they believed to be required for the tasks and (2) their ratings of their own competence in relation to the presumed requirements. It should be mentioned that some studies have indicated that the relationship between self-assessed and observed measures is weak [17,18]. However, we found that assessments of the knowledge gap varied substantially between the different groups of physicians included in our investigation.

Our results show that physicians need training in sickness insurance in a broad sense (Table 2). More than 50% indicated having a large or fairly large need for CPD about the roles of the various stakeholders, including themselves, and different types of compensation other than sick pay, as well as sickness insurance rules. This has not been shown in earlier studies, which have focused on more specific issues. Furthermore, four out of ten physicians reported the need for CPD regarding the following: how to assess patients' work capacity and work demands, as well as optimal length and degree of sickness absence; how to make plans of action for the sick-leave period, and how to handle conflicts with patients about sick leave. These observations support similar results found in earlier qualitative studies [10,13].

Previous research on physicians' knowledge and skills in insurance medicine has mainly considered the quality of the sick notes issued, not the physicians' own assessments of their knowledge regarding such tasks [5]. Clearly, investigations that consider physicians' own assessments of whether they want to learn more, and, if so, what they actually want to learn, would provide information that can be used to enhance future medical education and CPD.

It is noteworthy that most of the physicians in the present investigation reported that they had little need to improve their knowledge and skills in issuing sickness certificates. A systematic literature review as well as other studies [1,5] have established evidence that the quality of sick notes is often low from the perspective of the stakeholders who use such documents as a basis for determining whether patients are entitled to sickness benefits, that is, the patients' employers and the SIO. In one of those investigations [5], only 27% of the certificates provided clear assessments of both the medical disorder and the functional capacity of the patient, and 7% of the specialists' certificates were illegible. However, filling out sick notes was not considered to be very problematic by a majority of the physicians in our previous study [4], which suggests that the inferior quality of certificates is not a consequence of a lack of knowledge, but instead has other causes, such as low demands for quality from the SIOs [6]. On the other hand, a qualitative study performed in Norway [13] revealed difficulties associated with communication of assessments to the SIO, as indicated by this quotation from one of the interviewed physicians: "It's difficult to present it on a dotted line."

Our analyses of associations revealed two groups of correlated questionnaire items, and based on those, we created two indices that we designated the knowledge index and the skills index. Here, the word *knowledge *refers to what we call "know-what", or explicit knowledge, indicating information that can be verbalised and communicated to others. By comparison, a *skill *can be described as "know-how" or implicit knowledge [19]. For example, in a qualitative study [13], it was found that physicians described assessment of functional ability as being "in the back of our minds," and the authors called this phenomenon "tacit assessment."

The results of the present study indicated that the need for knowledge and skills was lower among those with longer professional experience measured as age, years in practice, or level of training (Table 5). This observation supports the Dreyfus and Dreyfus model [20] of development from novice to expert in a specific area, in which the expert relies on "pattern recognition" based on previous experiences, rather than explicit knowledge. However, in a study of the quality of sickness certificates [5], it was found that such documents more often lacked essential information when issued by specialists than when provided by those who were not yet specialists. Another study revealed that a larger number of sick notes were issued by more experienced physicians than by those with less experience [15]. We noted a small increase in the need for knowledge and skills in physicians who handled a large number of sickness certification cases and had a high frequency of problems in handling such documentation, as well as those who had regular contacts with the SIO (Table 5). Thus, more frequent handling of sickness certification does not imply a lower need for knowledge, as might be assumed.

Having a workplace policy on sickness certification was associated with less need for knowledge and skills. However, this must be interpreted with caution, since no conclusions regarding the direction of this association can be drawn from our results.

For both the knowledge and the skills index, the mean percentage of maximum was significantly higher for physicians working in primary care and psychiatry (Table 6), and was significantly lower for those working in surgery. To find plausible explanations for these inter-clinic disparities, we adjusted our results for a number of possible confounders, but the differences remained (Model 4, Table 6). These dissimilarities might be due to differences in the patient categories visiting the various types of clinics. Using that perspective, we can say that the sickness certification task differs depending on where a physician works, and thus the need for CPD ought to vary between physicians in different clinical settings and therefore might involve somewhat different aspects. Other interpretations of the results are also possible, for example, the idea of different sickness certification "cultures."

In a previous study of the same questionnaire data [4], we found that the level of problems experienced in sickness certification also varied substantially between clinical settings. In addition, reports of problems came from the same types of clinics/practices where many physicians indicated the need for further knowledge and skills in corresponding areas. For instance, a large number of physicians working in primary care had problems with this task, and they also expressed the need for more knowledge.

Physicians in primary care or occupational health services differed more from the reference group mean in the skills index than in the knowledge index (Model 4, Table 6). This could imply that they already had relatively high levels of knowledge, but they were confronted with more difficult cases and therefore needed relatively more skills than knowledge. Most of previous interventions (half or full day courses arranged by the SIO) have mainly been directed towards GPs.

For one of the items (E in Table 2), none of the correlations with the other items reached 0.500, probably due to few physicians wanting more knowledge or skills in issuing sick notes. Two other items (G and J) were only weakly associated with the other items, and hence they were not included in any of the indices. These two items were among those that the lowest percentage of physicians indicated they wanted to learn more about (Table 2).

### Implications for further research

Further research should be performed to elucidate the complex relationship between physician competence/experience and performance in insurance medicine, for example considering the quality of sick notes. Furthermore, studies are needed to explore the prerequisites for high quality in the sickness certification performed in different clinical settings.

### Implications for practice

It is essential that stakeholders in this area recognise that physicians feel they need not only knowledge, but also skills, in insurance medicine to deal with aspects such as assessments of work capacity and handling conflicts. Furthermore, the social insurance system lacks transparency in the sense that it does not allow physicians to fully understand their own responsibilities or those of other participants in the sickness certification process. Measures should be taken to improve physicians' competence in insurance medicine, and this should be done in pre- and postgraduate training, as well as in CPD.

## Conclusions

In this study, a majority of all physicians in most type of clinics and practices, not only primary care, stated that they needed more knowledge and skills to help them handle sickness certification. Nevertheless, few of the physicians felt they required more knowledge about issuing sickness certificates, which suggests that the poor quality of some sick notes is due to something other than the mere lack of knowledge and skills. Physicians working in primary care scored significantly higher than other physicians on the skills index, i.e. they had a larger need to develop their skills in sickness certification. CPD in insurance medicine should be strengthened to help increase the quality of management of sickness certification cases, in order to protect both the health and equity of patients.

## Competing interests

The authors declare that they have no competing interests.

## Authors' contributions

AL (PhD student), participated in the data collection, performed statistical analyses and drafted the manuscript. JH (PhD and statistician) participated in the statistical analyses and drafting of the manuscript. KA (Professor in Social Insurance) initiated the study, participated in the design and data collection of the study and drafting of manuscript. The manuscript has been read and approved by all authors.

## Pre-publication history

The pre-publication history for this paper can be accessed here:

http://www.biomedcentral.com/1471-2458/10/61/prepub
